# Dietary Patterns, Dietary Adequacy and Nutrient Intake in Adults Commencing Peritoneal Dialysis: Outcomes from a Longitudinal Cohort Study

**DOI:** 10.3390/nu16050663

**Published:** 2024-02-27

**Authors:** Kelly Lambert, Michele Ryan, Jade Flanagan, Georgie Broinowski, Maryann Nicdao, Jordan Stanford, Katrina Chau

**Affiliations:** 1 School of Medical, Indigenous and Health Sciences, University of Wollongong, Wollongong, NSW 2522, Australia; jf028@uowmail.edu.au (J.F.); gmb427@uowmail.edu.au (G.B.); jordan.stanford@newcastle.edu.au (J.S.); 2 Western Renal Service, Blacktown Hospital, Blacktown, NSW 2148, Australia; michele.ryan@health.nsw.gov.au (M.R.); maryann.nicdao@health.nsw.gov.au (M.N.); katrina.chau@health.nsw.gov.au (K.C.); 3 Blacktown Clinical School, School of Medicine, Western Sydney University, Blacktown, NSW 2148, Australia

**Keywords:** peritoneal dialysis, dietary pattern, dialysis, diet, nutrition, food, kidney failure, cohort study

## Abstract

(1) Background: Optimal dietary intake is integral to good health in people receiving peritoneal dialysis (PD). We investigated how dietary patterns, dietary adequacy and nutrient intake may change over time in people commencing PD. (2) Methods: Participants were attending the PD training unit for the commencement of peritoneal dialysis, aged ≥18 years and willing to complete food records. Misreporters were excluded from the analysis. Dietary intake was compared at PD commencement and at 12 months. Intake was also compared to reference standards. Dietary patterns were derived using principal component analysis. (3) Results: There were no significant changes between baseline and 12 months for grains, fruit, vegetables and meat. Dairy and added sugar intake was significantly lower (*p* = 0.01). The intake of energy and protein was adequate and did not change. There was a significant reduction in dietary phosphorus and calcium, and increased vitamin C intake. Three dietary patterns were identified: the ‘Bread and Cereal’ pattern; ‘Milk and Potatoes’ pattern; and the ‘Semi Vegetarian’ pattern. (4) Conclusions: In this longitudinal cohort study, the diet quality was suboptimal and there were limited changes in intake after the commencement of PD. Further exploration of how dietary patterns may impact outcomes and quality of life is warranted.

## 1. Introduction

Peritoneal dialysis (PD) is a form of renal replacement therapy (RRT) used by almost 3000 people in Australia with kidney failure [1]. Medical nutritional therapy is an essential component of care for PD [2]. The glucose load from dialysis increases the risk of high blood glucose levels, excess weight gain, and high triglycerides. Nutrients, including protein, are lost via dialysis and pathophysiological changes result in impaired nutrient absorption, early satiety, and gastrointestinal symptoms [3]. Disturbed appetite and satiety is also a noted feature of those established on PD [4]. 

From the limited evidence available on the diets of people undertaking PD, adherence to the renal diet prescription is estimated to range from 38% to 65.8% [5]. Dietary energy has been reported as inadequate in several small cross-sectional studies [6,7,8,9,10] and adequate in one [11]. Similarly, protein intake is often reported as inadequate [6,8,9,10,12,13]. From two studies that reported food group intake [9,14], data were incomplete and reported some, but not all, core food groups. In these studies, those on PD consumed adequate grain foods and inadequate fruits and vegetables [9,14]. The limitations of these studies are that they are cross sectional in nature and do not account for misreporting; therefore, they fail to quantify the accuracy of reported dietary intake in study participants. 

Few studies have also comprehensively documented the dietary and micronutrient intakes of people commencing PD or how intake may change over time. This information is critical as it is unclear if dietary intake improves with the removal of uremic toxins and fluid, and which foods or nutrients may change. Given the strong evidence for an association between healthy dietary patterns that are high in fruit, vegetables, legumes, cereals, wholegrains and fiber and a reduction in mortality in those with chronic kidney disease (CKD) [15], we aimed to explore nutrient and food group intake in a cohort of individuals commencing PD, and to describe if nutrient and food group intake changes over time after commencement. We also wished to explore and describe any dietary patterns that were evident in this group. 

## 2. Materials and Methods

This longitudinal cohort study is reported according to the STROBE guidelines for observational cohort studies [16]. The study utilizes data collected as part of a larger study examining the microbiota of patients commencing PD. 

In brief, all consecutive patients attending the home training unit for the commencement of peritoneal dialysis at the Western Sydney Local Health District Regional Dialysis Centre, Blacktown Hospital from December 2020 to July 2021 were invited by either KC or MN to participate in the larger study. Inclusion criteria for this study were people able to give informed consent, 18 years or older, were commencing PD, and were able to provide at least one day of food records. 

After written consent was obtained, participants were assessed by the site renal dietitian (MR). This dialysis service manages more than 975 dialysis patients [17], and is in a geographic location in metropolitan Sydney where >45% of residents born overseas and 45% speak a language other than English [18]. At baseline assessment, the dietary prescription provided was usual care [2,19], i.e., 1.0–1.2 g/kg, minimum 25 kcal/kg energy, <1000 mg dietary phosphate, <100 mmol sodium, 25–35 g fiber and food group intake consistent with the Australian Guide to Healthy Eating [20] except for dairy intake (1 serving per day and not 3–4 servings). Given the multicultural population serviced by the unit, to assist with accuracy of data completion, all participants were invited to photograph all meals, snacks and beverages consumed for at least one day (ideally three). For study participants who were unfamiliar with taking photos on their phone, the site dietitian (MR) provided basic training regarding how to take a photo and email or text message. These photographs were then sent to the renal dietitian to assist with analysis. Upon receival of photos, clarification was conducted to confirm that no meals, snacks or beverages were missed. Clarification was also obtained regarding foods eaten, portion sizes, fats used in cooking, condiments used and recipes. Clarification was obtained either in person when the patient attended the PD clinic or by phone if this was not possible. For those unable to take photos, dietary intake was assessed using a 24-h dietary recall. The use of plastic food models aided the determination of portion sizes.

In addition to dietary data, demographic information such as age, gender, PD prescription, comorbidities, BMI, ethnicity and symptom burden using the IPOS Renal [21] were also collected at baseline and 12 months by the site renal dietitian (MR) either in person or via phone (due to COVID-19 lockdown restrictions). Nutrition assessment was completed using the Subjective Global Assessment [22].

All food record data were entered manually into nutrient analysis software by two members of the research team (JF, GB). Foodworks Version 10 (Xyris Software Pty Ltd., Brisbane, Australia) with the AUSNUT 2011–2013 Australian Food Composition Database [23] was used for analysis. Where a specific food product was not available, a product with a similar nutrient composition was selected. Adequacy of nutrient intake was determined by comparing actual intake with the relevant dialysis specific nutrient recommended intake [2]. Nutrients not included in the dialysis specific guidelines included zinc, vitamin B6, vitamin C, potassium and fiber and were compared to the Australian Nutrient Reference Values Estimated Average Requirement [24]. Where this was not available, Average Intake (AI) was used. Reporting of dietary sodium intake was excluded from the study given the poor reliability of reported sodium intake [25]. For participants contributing more than one day of food records, average intake was calculated. 

In order to determine diet quality, all food group data output were converted manually to serves sizes described in the Australian Guide to Healthy Eating [20]. Food groups included grains, meat/poultry/fish, dairy, fruit, vegetables, alcohol and discretionary foods. Diet quality was adapted for dialysis by reducing adequacy to one serving of dairy per day to meet calcium and phosphorus targets for those undertaking PD. Discretionary food intake is not able to be computed in FoodWorks. Thus, added sugar was recorded instead and compared to the American Heart Foundation recommendations [26]. Adequate diet quality was defined as an intake of fiber ≥30 g per day, consumption of 2 servings of fruit and 5 servings of vegetables, 2–3 servings of meat and alternatives per day, 4 servings of grains and ≤1 serving of dairy per day, as well as meeting energy and protein recommendations (25–35 kcal/kg and 1–1.2 g/kg). We also calculated diet quality using three a priori plant-based diet indices (overall plant based diet index (PDI), healthy PDI, unhealthy PDI) [27]. The total score ranges from: PDI: 46–230; hPDI; 53–265; uPDI:51–255) (lowest to highest possible score). A higher PDI score represents greater consumption of all types of plant foods, regardless of healthiness. Both hPDI and uPDI consider food healthiness and ingredients from ‘core’ and ‘discretionary’ products are differentiated based on the Australian Health Survey: Users’ Guide, 2011–2013 – Discretionary Food List [28]. A higher hPDI score reflects greater consumption of healthy plant foods, mainly from the core foods group, and less of unhealthy plant foods. Conversely, a higher uPDI score indicates lower intakes of healthy plant foods and higher of unhealthy plant foods, particularly from discretionary sources.

Misreporters were excluded from the analysis using established cut off values [29]. Food records with a daily energy intake of below 500 kcal/2100 kJ and above 3500 kcal/14,700 kJ were excluded from the study. Normality was assessed using the Shapiro–Wilk test. Normally distributed data are reported as mean and standard deviation and nonnormal data as median and interquartile range. Descriptive statistics were used to describe baseline characteristics. Independent sample T-tests or Chi square tests were used to compare characteristics at baseline. A paired T-test or nonparametric equivalent was used to compare nutrient intake at commencement of PD and at 12 months. A one-sample T-test was used to compare nutrient or food group intake to nutrition benchmarks (Supplementary Table S1). McNemar’s test was used to determine differences in the proportion of individuals meeting nutrient targets at commencement of PD and at 12 months. SPSS (Version 28, SPSS, Chicago, IL, USA) was used for statistical analysis. A statistically significant *p*-value was set at ≤0.05.

To determine dietary patterns, Exploratory Factor Analysis using the principal component method was used to extract factors. Both baseline and follow-up data were included for the analysis. Orthogonal rotation was used as we wish to examine relationships among food groups. The varimax method of variance was used for the rotation of factors. Only factors with eigenvalues > 1.0 were extracted. Foods/food groups with a component load ≥ 0.50 were retained as this cut off is considered essential for interpreting practical significance [30]. A Kaiser–Meyer–Olkin (KMO) value of >0.5 and a significant (*p* < 0.05) Bartlett’s test of sphericity wase used to evaluate the factorability of the data [31]

The study was approved by the Western Sydney Local Health District Human Research Ethics Committee (Approval number 2019/ETH12569, 10 October 2019). 

## 3. Results

**Figure 1:**
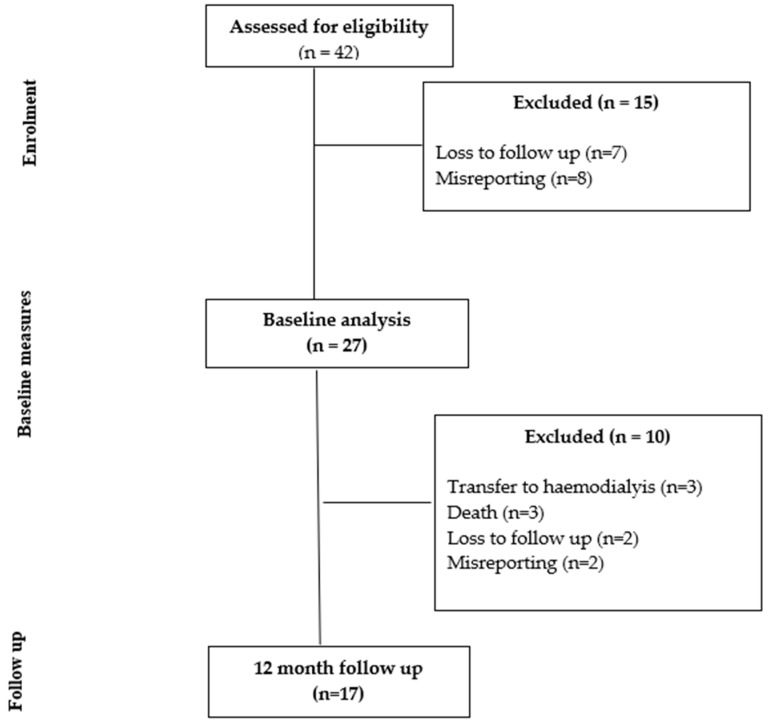
Study flow chart.

Of the 42 individuals recruited at baseline, 27 met the inclusion criteria for the baseline study. Of the 27 individuals included in the baseline study, 17 participants were seen at the 12 month follow up. Figure 1 shows the study flow diagram, with 10 participants lost at 12 months due to transfer to hemodialysis (n = 3), death (n = 3), misreporting (n = 2) and loss to follow up (n = 2). The characteristics of those assessed for eligibility and those who provided paired data at 12 months did not differ for age (*p* = 0.08), height (*p* = 0.60), weight (*p* = 0.27), BMI (*p* = 0.45), BMI class (*p* = 0.14) or presence of diabetes (*p* = 0.57). There were more males in the final analysis (*p* = 0.03, 56% when recruited, 88% in the final analysis). 

### 3.1. Characteristics of Participants

There were no significant differences in the characteristics of participants recruited at baseline and 12 months follow up for age, ethnicity, anthropometry or PD type (Table 1). Overall, participants experienced a wide range of symptoms, but few caused major impacts. The median total score was 12/60 (IQR: 6–19), and fatigue was rated as the symptom with the highest median score at baseline. Protein intake was inadequate at follow up with a median normalized protein catabolic rate of 0.79 (IQR: 0.67–1.04). Dialysis was >1.7 and considered adequate [32] at follow up with a median Kt/V of 1.79 (IQR:1.54–2.02).

### 3.2. Nutrient Intake 

Table 2 contains details of the nutrient intake for participants who completed assessments at baseline and 12 months. At baseline, energy intake was estimated to be a median of 26.9 (20.6–30.1) kcal/kg and 1.29 ± 0.46 g/kg for protein intake. This was aligned with or exceeded recommendations (25–35 kcal/kg and 1.0–1.2 g/kg) [2]. The intake of dietary phosphorus intake was excessive (1329 ± 345 mg) [2] and fiber intake was inadequate (23.8 ± 10.8 g/day) [24].

At 12 months, there was a slight but not significant increase in weight and BMI, but no significant change in the intake of energy (median 22.2 (IQR: 17.3–30.6 kcal/kg, *p =* 0.73)) or protein (1.00 ± 0.37 g/kg, *p =* 0.05). This was equivalent to a mean reduction of −767 ± 5160 kilojoules and −20 ± 44 g protein per day. Vitamin C intake increased from 97 ± 77 mg/day to 130 ± 118 mg (*p =* 0.002). There was a significant decrease in intake of saturated fat (−7 ± 11 g/day, *p =* 0.018), phosphorus (−323 mg, *p =* 0.01) and calcium (−176 mg, *p =* 0.031). Fiber and dietary potassium intake were not significantly different.

### 3.3. Diet Quality 

Diet quality did not change statistically between baseline and at 12-month follow up. Overall, diet quality was poor with excessive intake of grains at both time points (baseline 7.3 ± 4.0 servings and 12 months 5.6 ± 3.1 servings, *p =* 0.07), and inadequate intake of fruits (baseline 1.4 ± 1.3 servings and 12 months 2.1 ± 1.6 servings, *p =* 0.15) and vegetables (baseline 3.8 ± 2.3 servings and 12 months 4.0 ± 2.8 servings, *p =* 0.83). The intake of added sugar was below recommendations of <36 g per day and this was reduced further at 12 months (mean difference 10 ± 25 g, *p <* 0.001). Notably, while mean dairy intake was not significantly lower at 12 months (mean intake 0.6 ± 0.7 servings per day, *p =* 0.08), the proportion of participants who meeting dietary phosphorus targets improved (*p =* 0.03) (Table 3). No participants achieved the minimum 800–1000 mg of calcium per day; the mean intake at follow up was 495 ± 247 mg per day. Other changes in diet quality included an Increase in the proportion of participants achieving adequate fiber (*p =* 0.005), and proportion achieving daily added sugar targets (*p =* 0.001). No changes in diet quality scores were seen including the overall PDI, healthy PDI or unhealthy PDI. 

### 3.4. Dietary Patterns 

Three distinct dietary patterns were identified, which explained 74.808% of the variance in dietary intake (Table 4). No cross loading of patterns was apparent. Pattern 1 was the most dominant pattern and was characterized by a high intake of whole and refined grains and labeled the ‘breads and cereals’ pattern. Pattern 2 was labelled the ‘milk and potatoes’ pattern and was dominated by a high intake of milk and potato products. Pattern 3 was rich in legumes and red orange vegetables and low in fruit and labeled ‘semi vegetarian’. The Bartlett’s test of sphericity indicated that the correlation matrix was not random, χ^2^(df = 105) = 2626.82, *p* < 0.0001, and the KMO statistic was 0.51, suggesting it was mediocre and below the ideal standard of 0.6 for conducting factor analysis. Despite this, it was determined that the correlation matrix was appropriate for factor analysis.

## 4. Discussion

There are several major findings of this study that are novel regarding the nutrient and dietary intake of people commencing peritoneal dialysis. Firstly, the intake did not change substantially 12 months after commencement of dialysis. Second, the energy intake declined but did not change significantly after the commencement of PD. Protein intake also did not differ over time. Finally, the diet quality of participants was overall suboptimal, with an inadequate intake of fruit, vegetables and fiber, and excessive intake of breads and cereals. However, the intake of added sugar and dairy did improve over time. Three distinct dietary patterns were also identified.

One explanation for the findings of generally adequate energy and protein intake may be the adequate nutritional status, short dialysis vintage and relatively low intensity of symptoms involving appetite and dietary intake at baseline. Participants reported the major symptom at commencement was fatigue, and symptoms such as nausea, vomiting and poor appetite were not troublesome or only slightly problematic. This contrasts with other studies of symptom burden in people undertaking PD, where the loss of appetite was more common and more severe [33,34,35]. In the present study, we were unable to collect repeat POS renal symptom burden scores at 12 months due to COVID-19 pandemic restrictions. As per the recent recommendations from the KDOQI Controversies conference on managing the symptom burden associated with maintenance dialysis [36], routine screening and surveillance of symptom burden using validated tools is recommended. In particular, proactive questioning about symptom burden and whether these symptoms may impact dietary intake would be useful.

In contrast to other studies of people undertaking PD, mean dietary fiber intake was ~24 g per day. While suboptimal, this amount is aligned with findings by Azarnoush et al. who reported mean intake of 24–28 g/day [13] and significantly higher than reported in several previous studies that were ~10–15 g per day [9,37]. Adequate dietary fiber is highly beneficial for people undertaking PD, and inadequate intake is associated with constipation as well as negative outcomes, including PD catheter dysfunction (flow failure) [38,39], increased accumulation of uremic toxins [40], susceptibility to peritonitis [41] and mortality [42]. Strategies that focus on improving diet quality, in addition to liberalizing previous dietary restrictions or beliefs about fruit and vegetable intake and ensuring adequate fluid and physical activity, are critical to improving the management of constipation. Appropriate pain management with the minimal use of opioid medications, which may contribute to constipation, is important. This has been highlighted as a key barrier in many with kidney failure, as polypharmacy is common and many individuals undertaking dialysis are reluctant or unable to afford additional medications [43]. 

The findings of this study regarding dietary patterns are informative and yield more information than isolated reporting of nutrients or foods. For example, the low number of individuals meeting fruit intake recommendations in this study (24% and 47%) are consistent with those from the most recent Australian Health Survey [44], where 44.1% of adults met the recommendation. In contrast, adequate vegetable intake appeared higher in this sample (baseline 35% and 41% at follow up) compared to 6.5% of adults in the Australian Health Survey [44]. While sample size was small, there was still sufficient evidence that eating habits are not uniform in this group. Dietary education strategies require individualized advice to achieve improvements in nutrient and diet quality targets, particular those regarding fiber, fruit and vegetable intake. Given the extensive benefits of adequate fruit and vegetable intake in those with CKD on acidosis, weight management and mortality [45] and the move to food-based dietary prescriptions for people with CKD [2], future research should explore the impact of novel educational strategies for people with CKD to effectively increase the intake of these foods. This may include education on ‘plant-based diets’ [46]. 

One of the challenges unique to dietary research is that food frequency questionnaires are cuisine and culture specific. Unlike questionnaires about medications or symptoms, dimension reduction techniques in dietary pattern analysis require subjective judgments about grouping of similar foods, and a universal FFQ that can be applied to all undertaking PD is not possible. Given this limitation, it would be useful in future for researchers to include the reporting of core food groups and not just nutrients to enable comparisons of core foods as well as nutrient intake across studies. 

The strengths of this work are the comprehensive nutrient and food group reporting as well as the identification of misreporters. This enables a more accurate picture of intake to be captured alongside symptom burden. In addition, all dietary data was captured by the same qualified dietitian and verified using multiple methods. The limitations were the small sample size dominated by males, the relatively short duration of follow up and use of diet history data, which only captures intake in the short term. Calories absorbed from dialysate were not included in energy intake calculations, and differences in intake between PD types were not explored due to the small sample size. Given the clinically important increase in number who were obese at 12 months, future research should investigate body composition changes in more detail. Future research with a larger sample size would be useful as the sampling adequacy for principal component analysis was considered borderline. The use of a comprehensive food frequency questionnaire would also enable more explicit and descriptive dietary patterns to be identified. Exploring changes in appetite, satiety and bowel function would also be informative given recent studies on the prevalence of constipation [47], gastrointestinal symptoms and eating dysfunction in PD [48]. Future research exploring the dietary beliefs and food preferences of those undertaking PD would also be useful. It is unclear if reductions in dairy and sugar and the low intake of fruits and vegetables were due to symptoms, dietary misconceptions or lack of knowledge or other unknown factors. 

## 5. Conclusions

Nutrient intake and diet quality were largely unchanged between the commencement of PD and at 12 months in this study. Given that the overall diet quality was suboptimal, and few studies have explored dietary patterns in this patient group, additional research is required to explore how advice to improve dietary patterns may be used to improve health outcomes and quality of life. 

## Figures and Tables

**Table 1:** Characteristics of participants.

Participants Characteristics	Baseline (n = 27)	Follow Up (n = 17)	*p*-Value
Demographics			
Age, mean (SD)	63.04 (13.37)	66.24 (12.59)	0.43
Female, n (%)	8 (30%)	2 (12%)	0.17
Ethnicity, n (%)			0.90
Asian	10 (37%)	7 (41%)	
Indigenous Australian	0	0	
Caucasian	8 (30%)	2 (11%)	
Pacific Islander	4 (15%)	4 (24%)	
Middle Eastern	5 (18%)	4 (24%)	
Anthropometry			
Weight (kg), mean (SD)	77.91 (16.53)	78.91 (15.71)	0.84
BMI (kg/m^2^), mean (SD)	27.14 (3.74)	27.76 (4.7)	0.26
BMI Class (kg/m^2^), n (%)			0.05
Underweight (<18.5)	0	0	
Normal (18.5–24.9)	8 (30%)	5 (30%)	
Overweight (25–29.9)	11 (40%)	6 (35%)	
Obese (>30)	8 (30%)	6 (35%)	
Diabetes, n (%)	11 (41%)	8 (47%)	0.68
PD type, n (%)			0.89
CAPD/APD	18 (66%)	11 (65%)	
IPD	9 (33%)	6 (35%)	
Food intake confirmed with photos	23 (85%)	15 (88)	0.77
Nutritional Status			0.16
Well nourished (SGA = A)	13 (77)	15 (88)	
Mild or moderately malnourished (SGA = B)	4 (24)	1 (6)	
Malnourished (SGA = C)	0 (0)	1 (6)	
nPCR	-	0.79 (IQR: 0.67–1.04)	-
Membrane transporter status			
High	-	5 (29)	-
High average	-	4 (24)	
Low average	-	7 (41)	
Low	-	1 (6)	
Dialysis Adequacy Kt/V	-	1.79 (IQR:1.54–2.02)	-
Biochemistry at follow up	-	-	-
Sodium (mmol/L)	-	134 (3.5)	-
Potassium (mmol/L)	-	4.3 (0.8)	-
Bicarbonate (mmol/L)	-	26 (3.3)	-
Urea (mmol/L)	-	22 (6.2)	-
Creatinine (umol/L)	-	744 (197)	-
Calcium (corrected) (mmol/L)	-	2.5 (0.2)	-
Phosphate (mmol/L)	-	1.67 (0.47)	-
Magnesium (mmol/L)	-	0.88 (0.15)	-
Albumin (g/L)	-	29.4 (3.0)	-
Hemoglobin (g/L)	-	107 (14)	-
IPOS score, median (IQR)			
Pain	1.00 (0.00–2.00)	-	
Shortness of breath	0.50 (0.00–1.00)	-	
Weakness or lack of energy	1.50 (0.00–2.25)	-	
Nausea	0.00 (0.00–1.00)	-	
Vomiting	0.00 (0.00–0.00)	-	
Poor appetite	0.50 (0.00–2.00)	-	
Constipation	1.00 (0.75–2.00)	-	
Sore or dry mouth	1.00 (0.00–2.00)	-	
Drowsiness	1.00 (0.00–2.00)	-	
Poor mobility	1.00 (0.00–2.00)	-	
Itching	1.00 (0.00–2.00)	-	
Difficulty sleeping	1.00 (0.00–2.00)	-	
Restless legs	0.00 (0.00–1.00)	-	
Changes in skin	0.50 (0.00–2.00)	-	
Diarrhea	0.00 (0.00–0.00)	-	
Total Score	12 (6–19)	-	

Legend: CAPD: Continuous Automated Peritoneal Dialysis; APD: Automated Peritoneal Dialysis; IPD: Incremental Peritoneal Dialysis; IPOS Renal scores not available at 12 months; SGA: Subjective Global Assessment; nPCR: normalized Protein Catabolic Rate; Kt/V: mass of urea removed. Full details of PD regimen included in Supplementary Table S2.

**Table 2:** Nutrient and dietary intake at baseline and 12 months.

	Baseline (n = 17) Mean (SD)	Follow Up (n = 17) Mean (SD)	*p* Value *a*	Mean Difference (SD)	*p* Value *b*
Demographic characteristics					
Weight (kg)	77.00 (13.29)	78.91 (15.71)	0.23	1.91 (6.27)	-
BMI (kg/m^2^)	27.14 (3.74)	27.76 (4.70)	0.26	0.62 (0.53)	-
Macronutrients					
Energy (kJ) median (IQR)	8459 (6763–9366)	6665 (6253–10,012)	0.57	–767 (5160)	-
Energy (kcal/kg/day) (median (IQR)	26.9 (20.6–30.1)	22.7 (17.3–30.6)	0.73	–1.8 (12.1)	0.80
Protein (g)	97 (32)	77 (24)	0.07	–20 (43)	-
Protein (g/kg)	1.29 (0.46)	1.00 (0.37)	0.05	–0.29 (0.57)	0.25
Fat (g)	77 (28)	64 (26)	0.20	−12 (38)	-
Sat fat (g)	28 (11)	20 (8)	0.02	−7 (11)	--
CHO (g)	204 (79)	186 (72)	0.44	−18 (84)	-
Micronutrients					
Phosphorus (mg)	1329 (345)	1006 (344)	0.01	−323 (448)	0.03
Potassium (mg)	2955 (933)	2589 (1013)	0.33	−366 (1509)	0.81
Calcium (mg)	671 (320)	495 (247)	0.03	−176 (304)	0.01
Zinc (mg)	9.87 (4.07)	8.94 (3.69)	0.41	−1 (5)	0.02
Vitamin B6 (mg)	1.65 (0.87)	2.07 (1.45)	0.34	0 (2)	0.11
Vitamin C (mg)	97 (77)	130 (118)	0.41	33 (156)	0.002
Fiber (g)	23.8 (10.8)	24.1 (10.1)	0.93	0.29 (13.5)	0.01
% kJ from fiber	2.35 (0.76)	2.68 (0.83)	0.17	0.33 (0.94)	-
% kJ from CHO	41.30 (9.21)	42.44 (8.62)	0.66	1.14 (10.60)	-
Core food groups					
Grain serves	7.3 (4.0)	5.6 (3.1)	0.07	−1.7 (3.7)	0.08
Fruit serves	1.4 (1.3)	2.1 (1.6)	0.15	0.7 (1.9)	0.17
Vegetable serves	3.8 (2.3)	4.0 (2.8)	0.83	0.2 (3.4)	0.32
Dairy serves	1.1 (1.0)	0.6 (0.7)	0.08	−0.4 (1.0)	0.03
Meat/ alternative serves	3.0 (1.3)	2.3 (1.1)	0.11	−0.7 (1.6)	0.28
Alcohol standard serves	0.3 (0.6)	0.41 (1.0)	0.21	0.2 (0.5)	1.00
Added sugar (g)	24 (23)	14 (14)	0.11	−10 (25)	<0.001
Diet Quality Indices (Range of possible scores: PDI: 46–230; Healthy PDI; 53–265; Unhealthy PDI:51–255)					
Plant-Based Diet Index (PDI)	137.9 (9.7)	137.9 (8.5)	1.00	0.2 (12.4)	-
Healthy PDI	160.4 (14.3)	158.9 (13.0)	0.60	−1.4 (11)	-
Unhealthy PDI	154.8 (16.2)	153.7 (19.2)	0.67	−1.1 (10.2)	-

*a*: *p* value for difference between timeframes of baseline and 12 months. *b: p* value for difference in the proportion meeting recommendations.

**Table 3:** Proportion of participants meeting diet quality targets at baseline and 12 months (n = 17, paired data).

Nutrient/Food Target	Proportion Meeting Targets at Baseline n (%)	Proportion Meeting Targets at 12 Months n (%)	*p* Value
Adequate Energy (25–35 kcal/kg/day)	10 (59)	8 (47)	0.49
Protein (1–1.2 g/kg)	7 (41%)	5 (30%)	0.25
Phosphorus (≤1000 mg/day)	2 (12%)	8 (47%)	0.03
Calcium (800–1000 mg/day)	5 (29%)	0 (0%)	0.01
Adequate zinc (≥12 mg/day)	4 (24%)	3 (18%)	0.02
Adequate Vitamin B6 (≥1.4 mg/day)	13 (76%)	11 (65%)	0.11
Adequate Vitamin C (≥30 mg/day)	16 (94%)	14 (82%)	0.002
Adequate fiber (≥30 g/day)	3 (18%)	7 (41%)	0.005
Adequate grains (4 servings/day)	15 (88%)	10 (59%)	0.08
Adequate fruit (2 servings/day)	4 (24%)	8 (47%)	0.17
Adequate vegetables (5 servings/day)	6 (35%)	7 (41%)	0.32
Adequate dairy (≤1 servings/day)	11 (41%)	9 (53%)	0.63
Meat and alternative (2–3 servings/day)	8 (47%)	4 (24%)	0.28
Alcohol (≤4 standard drinks/day)	17 (100%)	17 (100%)	1.00
Added sugar (<10% total energy or <36 g/day)	13 (76%)	16 (94%)	0.001

**Table 4:** Exploratory factor analysis of dietary patterns with factor loadings.

Food Component	Pattern 1 Breads and Cereals	Pattern 2 Milk and Potatoes	Pattern 3 Semi Vegetarian
Refined grains	0.977		
Wholegrains	0.976		
Milk		0.705	
Potato products		0.676	
Red/orange vegetables			0.761
Fruit			−0.495
Legumes			0.372
Seafood			
Nuts and seeds			
Yoghurt			
Dark green vegetables			
Cheese			
Poultry			
Eggs			
Alcoholic drinks			
Amount (%) of variance explained	13.433	7.135	6.925

Coefficients with values <0.50 are not shown. The higher the coefficient, the stronger the correlation.

## Data Availability

The data presented in this study are available on request from the corresponding author. The data are not publicly available due to privacy.

## Supplementary Materials

The following supporting information can be downloaded at: https://www.mdpi.com/article/10.3390/nu16050663/s1, Table S1: Recommended nutrient intake for adults on peritoneal dialysis using a 70 year old reference person; Table S2: PD regimen at baseline and 12 month follow up. References [2,24,26,49] are cited in supplementary file.
