# Supplementary material for: Dietary Patterns, Dietary Adequacy and Nutrient Intake in Adults Commencing Peritoneal Dialysis: Outcomes from a Longitudinal Cohort Study
Source: Nutrients. 2024 Feb 27;16(5):663. doi: 10.3390/nu16050663 (PMC10935117; doi:10.3390/nu16050663)
Supplement: Supplementary file 1 [file nutrients-16-00663-s001.zip › nutrients-2841658-supplementary.pdf]

Supplementary Table S1. Recommended nutrient intake for adults on peritoneal dialysis using a 70 year old reference person.

Nutrients	Nutrient target	Benchmark	Reference
**Energy**	105-145kJ/kg IBW/day	126kJ/kg	KDOQI [1]
**Protein**	1.0-1.2g/kg IBW/day	1.1g/kg	KDOQI [1]
**Fat**	No recommendation	N/A	-
**Carbohydrate**	No recommendation	N/A	-
**Fibre**	Female 25g/day (AI)<br>Male 30g/day (AI)	30g/day	NHMRC NRV [2]
**Zinc**	Females: 6.5mg/day (EAR)<br>Males: 12mg/day (EAR)	12mg/day	NHMRC NRV [2]
**Calcium**	800-1000mg/day	900mg	KDOQI [1]
**Phosphorus**	<1000mg/day	<1000mg	KDOQI [1]
**Potassium**	2800mg/day	2800mg	KDOQI [1]
**Sodium**	<2300mg/day (UL)	2300mg	KDOQI [1]
**Vitamin C**	Females: 30mg/day (EAR)<br>Males: 30mg/day (EAR)	30mg	NHMRC NRV [2]
**Vitamin B6**	Females: 1.3mg/day (EAR)<br>Males: 1.4mg/day (EAR)	1.4mg	NHMRC NRV [2]
**Food groups**			
**Grains (bread, cereals, legumes, rice, pasta)**	Females: 3 serves<br>Males: 4 serves	4 serves	AGTHE [3]
**Protein (meat, poultry, fish, tofu, eggs) #**	Females & Males: 2-3 serves	2.5 serves	Adapted from KDOQI and AGTHE [1,3]
**Dairy (cheese, yoghurt, milk, and alternatives) #**	Females & Males: 1-2 serves	1.5 serve	Adapted from KDOQI and AGTHE [1,3]
**Fruit**	Females: 2 serves<br>Males: 2 serves	2 serves	AGTHE [3]
**Vegetables**	Females: 5 serves<br>Males: 5 serves	5 serves	AGTHE [3]
**Alcohol**	<4 standard drinks	≤4 standard drinks	AGTHE [3]
**Discretionary – Added Sugar (g)**	<36g (UL)	≤36g	American Heart Foundation [4]

Legend: KDOQI: Kidney Disease Outcomes Quality Initiative; NRMHC NRC: National Health and Medical Research Council Nutrient Reference Values; ADG: Australian Dietary Guidelines

The reference person selected for fibre, vitamin and mineral intake was a 71 year old male (median intake of the study population)

Serves of protein foods and dairy intake are adapted to meet the protein, calcium and phosphorus needs of people undertaking dialysis

References:

1. Ikizler, T.A.; Burrowes JD; Byham-Gray LD, e.a.; KDOQI Nutrition in CKD. Guideline Work Group. KDOQI Clinical Practice guideline for nutrition in CKD: 2020 update. *Am J Kidney Dis* **2020**, *76*, S1-S107.
2. National Health and Medical Research Council. *Nutrient Reference Values for Australia and New Zealand*; 2020.
3. National Health and Medical Research Council. Australian Guide to Healthy Eating. Available online: https://www.nhmrc.gov.au/adg (accessed on 10 January 2024).
4. American Heart Association. AHA Sugar Recommendation. Available online: https://www.heart.org/en/healthy-living/healthy-eating/eat-smart/sugar/how-much-sugar-is-too-much#:~:text=AHA%20Sugar%20Recommendation&textMen%20should%20consume%20no%20more,or%20100%20calories)%20per%20day (accessed on 11 December 2023).

Supplementary Table S2. PD regimen at baseline and 12 month follow up

ID	Initial prescription	Follow up prescription	Dry day at FU (Yes = 1 No = 0)
107	Full CAPD, 4x2L, mostly 3x1.5, 1x2.5	Full	0
109	Incremental, 1x2L, 1.5%	Full,APD	1
110	Incremental,,2x2L	Incremental, 2x2L	part-dry
111	Full CAPD, 4x2L, mostly 3x1.5, 1x2.5	Full	0
115	Incremental,APD: 3 x 2L 2.5% over 8.5 hrs	Incremental,,APD 3x2.3L	1
117	Full, APD: 4 x 2L	Full, APD	0
119	Full,commenced 3x2.5% 2L, 1x1.5% 2L	Full	0
122	Full, APD: 4 x 2L, mix of 2.5 and 1.5%	Full, APD	1
125	Incremental,, APD: 4x1.8L, mix of 1.5 and 2.5%	Full, APD	1
127	Full,4x2L: 3x2.5%, 1x1.5%	Full	0
137	Incremental,,1x2L, alternating b/w 1.5 and 2.5%	Incremental, 2x1.5L	part-dry
139	Incremental,1 x 2L, 2.5%	Incremental, 1x2L	1
141	Incremental,, 1x2L 7.5%	Full	0
142	Incremental,, 3 bags: 2 x 2L 2.5%, 1 x 7.5% 2L for nocturnal dwell	Full	0
144	Incremental, APD: 4 x 1.8 L 2.5%	Incremental, APD: 4x1.8L	1
145	Incremental, 3 x 2L: 2 x 2.5%, 1 x 1.5%	Incremental,3x2L	part dry
147	Incremental,1 x2L 1.5%	Full	0
